# Assessment of Local Dynamic Stability in Gait Based on Univariate and Multivariate Time Series

**DOI:** 10.1155/2019/6917658

**Published:** 2019-07-25

**Authors:** Henryk Josiński, Adam Świtoński, Agnieszka Michalczuk, Piotr Grabiec, Magdalena Pawlyta, Konrad Wojciechowski

**Affiliations:** ^1^Institute of Informatics, Silesian University of Technology, Akademicka 16, 44-100 Gliwice, Poland; ^2^Centre for Research and Development, Polish-Japanese Academy of Information Technology, Aleja Legionów 2, 41-902 Bytom, Poland

## Abstract

The ability of the locomotor system to maintain continuous walking despite very small external or internal disturbances is called local dynamic stability (LDS). The importance of the LDS requires constantly working on different aspects of its assessment method which is based on the short-term largest Lyapunov exponent (LLE). A state space structure is a vital aspect of the LDS assessment because the algorithm of the LLE computation for experimental data requires a reconstruction of a state space trajectory. The gait kinematic data are usually one- or three-dimensional, which enables to construct a state space based on a uni- or multivariate time series. Furthermore, two variants of the short-term LLE are present in the literature which differ in length of a time span, over which the short-term LLE is computed. Both a state space structure and the consistency of the observations based on values of both short-term LLE variants were analyzed using time series representing the joint angles at ankle, knee, and hip joints. The short-term LLE was computed for individual joints in three state spaces constructed on the basis of either univariate or multivariate time series. Each state space revealed walkers' locally unstable behavior as well as its attenuation in the current stride. The corresponding conclusions made on the basis of both short-term LLE variants were consistent in ca. 59% of cases determined by a joint and a state space. Moreover, the authors present an algorithm for estimation of the embedding dimension in the case of a multivariate gait time series.

## 1. Introduction

Stability means the ability to return to a stable state after having been subjected to some form of perturbation. Focusing on gait, if infinitesimally small perturbations, naturally occurring tiny variations in the walking surface and/or natural noise in the neuromuscular system, are concerned, then the ability of the locomotor system to keep the gait smooth by attenuating them is called local dynamic stability (LDS) [1]. The aforementioned disturbances are the cause of slightly different conditions at the beginning of successive strides. As a consequence, the LDS can be assessed using a measure of the extreme sensitivity to initial conditions.

Gait stability is of great importance for older people who are considered prone to falls. It requires constantly working on different aspects of LDS assessment method, which is derived from the dynamical systems theory. The method is based on a trajectory in a state space which is reconstructed from time series generated by a dynamical system. The dynamical properties of a system in the true state space are preserved under the reconstruction process, which enables to analyze the system's behavior using the reconstructed trajectory, with particular emphasis on system's sensitivity to initial conditions.

The authors intended to investigate how a state space structure affects the LDS. The input data for constructing a state space were time series describing the movement at a single joint. The authors created state spaces on the basis of one- or three-dimensional time series for hip, knee, and ankle joints separately and used the reconstructed trajectory for the LDS assessment according to the approach briefly described in the next section.

## 2. Materials and Methods

### 2.1. Theoretical Background

A symptom of extreme sensitivity to initial conditions is the exponential rate of divergence of trajectories from their starting points which are located in a state space very close to each other. This rate, which is called the largest Lyapunov exponent (LLE), is defined as follows:

(1) $$\begin{array}{c} \lambda_{1}=\underset{t⟶\infty }{\lim}\frac{1}{t}\;\cdot \;\ln\left[\frac{d\left(t\right)}{d\left(t_{0}\right)}\right], \end{array}$$

where *t*_0_ is the initial time instant and *d*(*t*) represents a distance between corresponding points on initially nearby trajectories at any time instant *t*. From the perspective of gait analysis, a positive LLE value indicates locally unstable behavior (i.e., trajectories diverge; however, due to the presence of the attractor the distance between them cannot grow without limit). The higher the LLE, the greater the system's sensitivity to extremely small perturbations during gait and thus the lower the LDS. The LLE, which estimates the local stability immediately after a potential perturbation, is called the short-term largest Lyapunov exponent. The short-term LLE is computed over a time span of a length either corresponding to one step [2–4] or one stride [1, 5, 6] using the Rosenstein algorithm [7]. The idea behind this method is that pairs of segments of the state space trajectory reconstructed on the basis of experimental data repeatedly imitate two initially neighboring trajectories, which makes it possible to trace the divergence of them. A comprehensive and precise description of the component methods leading finally to the determination of LLE was included in the work of Perc [8].

A state space structure is an important aspect of the LDS assessment. The reconstruction procedure is based on two parameters: *time delayτ* (*reconstruction delay*, *lag*) and *embedding dimensionm*. For a time series, which is composed of *N* points {*x*_1_, *x*_2_,…, *x*_*N*_}, an *m*-element vector of delay coordinates of the point **x**_*i*_ on the reconstructed trajectory is given by [*x*_*i*_, *x*_*i*+*τ*_, *x*_*i*+2·*τ*_,…, *x*_*i*+(*m* − 1)·*τ*_], where *i*=1,2,…, *M*=*N* − (*m* − 1) · *τ* (the reconstructed trajectory consists of *M* points) [9].

For a multivariate time series, which is composed of *K* univariate time series of equal length *N*, the reconstruction parameters are defined by a time delay vector [*τ*_1_, *τ*_2_,…, *τ*_*K*_] and an embedding dimension vector [*m*_1_, *m*_2_,…, *m*_*K*_]. Therefore, the state space dimensionality *m* is a sum of all *m*_*k*_, *k*=1,2,…, *K*, and the *m* delay coordinates of the point **x**_*i*_ form a vector [*x*_1,*i*_, *x*_1,*i*+*τ*_1__,…, *x*_1,*i*+(*m*_1_ − 1)·*τ*_1__, *x*_2,*i*_, *x*_2,*i*+*τ*_2__,…, *x*_2,*i*+(*m*_2_ − 1)·*τ*_2__,…, *x*_*K*,*i*_, *x*_*K*,*i*+*τ*_*K*__,…, *x*_*K*,*i*+(*m*_*K*_ − 1)·*τ*_*K*__], where $i=1,2,\ldots ,M=N–\underset{k}{\max}\left\{\left(m_{k}–1\right)\cdot \tau_{k}\right\}$ [10, 11].

### 2.2. Review of the Previous Work

Concentrating on the importance of analyzing LDS and utilizing its results, some other pieces of research deserve a mention, e.g., Terrier et al. [12] investigated LDS in patients with chronic impairments after foot and ankle injuries, Bruijn et al. [13] discussed the relationship between gait stability and arm swing, and Dingwell and Marin [5], as well as England and Granata [6], analyzed the influence of gait speed on LDS. Several studies [14, 15] indicate that LDS is associated with the fall risk. Moreover, LDS may be used as a potential fall predictor to differentiate fall-prone adults [16]. LDS turned out to be sensitive to age-related degeneration [2]. The authors of [3] show that dance training might improve LDS of normal walking of the elderly. Another important aspect of LDS is its suitability for monitoring of geriatric or neurological pathologies in their early phases [2]. Comprehensive reviews of measures assessing the stability of human locomotion were prepared by Hamacher et al. [17], Bruijn et al. [18], and Van Emmerik et al. [19].

The discussion of state space structures in the context of motion data was initiated by Gates and Dingwell [20] who focused on univariate time series based on Euler angles describing rotational motion of a shoulder. The authors conclude that the comparison between outcomes for different state spaces should be made with caution; however, the trends identified in the analyzed data remain relevant. Besides, the authors do not recommend the tested PCA-based reduction of a state space dimensionality. In [21], a multivariate time series, which represented movement at hip, knee, and ankle joints in the sagittal plane, was applied to analyze quiet standing balance. As far as the total embedding dimension for a multivariate time series is concerned, Vlachos and Kugiumtzis [10] presented two modified variants (FNN1 and FNN2) of the false nearest neighbors method [22], which were adjusted to a multivariate time series. According to FNN1, the same embedding dimension is applied to all the component time series. The FNN2 method is an exhaustive algorithm. The third method proposed in [10] was based on the criterion of prediction error minimization (PEM), whereas Zhang et al. [23] suggested applying a maximal joint entropy criterion. The consequences of a fixed time delay and/or a fixed embedding dimension were investigated by van Schooten et al. [4]. Hamacher et al. [2] evaluated multiple state space definitions differing in signal type (linear acceleration and angular velocity), signal dimension (one-dimensional and three-dimensional), and location of an inertial sensor (trunk and forefoot). Piórek et al. [24] used a quaternion-based interpretation of body segments' rotations and replaced a multivariate time series of Euler angles by a quaternion angle time series. Moreover, they showed a correlation between LLE values computed for time series consisting of (1) quaternion angles and (2) joint angles in a group of young individuals for hip, knee, and ankle joints in different variants of walking speed and ground inclination. The same set of experimental data was also analyzed using a new quaternion-based variant of the approximate entropy measure [25]. A systematic review of methodological approaches of the LLE quantification was prepared by Mehdizadeh [26].

It should also be pointed out that the range of applications of the LLE as a measure of sensitivity to infinitesimal changes in initial conditions goes beyond the gait analysis. For instance, Jagrič et al. [27] analyzed the irregularity in short electrocardiographic (ECG) recordings in a similar manner to predict successful defibrillation in patients with ventricular fibrillation. A higher level of irregularity was interpreted as an indicator of patients who may be subjected to effective defibrillation.

### 2.3. The Goal of the Research

As mentioned above, the examined state space structures were constructed on the basis of time series built of joint angles at hip, knee, and ankle joints. Three time series, which are related to the given joint, represent specific types of movement in sagittal, frontal, and transverse planes. For instance, movements at hip joint are called flexion/extension, abduction/adduction, and internal/external rotation, respectively. However, analysis of human gait focuses often on the sagittal plane to which the vast majority of the work during gait is assigned (ca. 74%, 85%, and 93% in case of hip, knee, and ankle joints, respectively) [28]. All the planes can be included using a state space based on a multivariate times series. At the previous research stage, which was extensively described in [29], the authors only used one state space that was based on multivariate time series composed of experimental data recorded in the CAREN extended environment (http://www.motekforcelink.com/product/caren/). Various experiments' scenarios (i.e., variants of gait) were proposed which differed from each other with respect to walking speed, platform slope, and optional external perturbation. The results presented here are based on the same set of experiments. However, this time both the comparison of the LDS in three pairs of the “opposed” scenarios (i.e., gait variants which differ in one of the aforementioned aspects) and the statistical analysis were made for ankle, knee, and hip joints separately. Moreover, two additional structures of a state space were taken into consideration. The state spaces based either on a multivariate times series or on a univariate time series, which represents joint angles in the sagittal plane, will be described in Section 2.4.

Finally, three state space structures, which were constructed for each joint separately, were used to verify if the differences in LLE values between the opposed scenarios are significant for individual joints.

The authors also present a modification of the LDS computation method, i.e., an algorithm for estimation of one of its crucial parameters, embedding dimension, for the case of a multivariate gait time series, in which the parameter is not estimated for each of the component time series separately, but holistically.

The following research questions are addressed in the paper:

- Are there any significant differences in the local dynamic stability between compared gait variants, which can be revealed using the individual state spaces?
- Is the predominant role of sagittal plane preserved in a state space which is based on a multivariate time series?
- Does the length of the time span, over which the short-term LLE is computed, influence the difference in the local dynamic stability between compared gait variants?

### 2.4. The Research Procedure

The research procedure was composed of the following steps:

1. Data acquisition and preprocessing.
2. Estimation of the reconstruction parameters.
3. Trajectory reconstruction.
4. Estimation of the short-term LLE, taking into consideration both the aforementioned time span variants.

Inspired by reports in the literature, the authors decided to incorporate three different state space structures into research. The *UniS* state space is reconstructed on the basis of a univariate time series describing a movement at a joint in the sagittal plane. The next two spaces—*MultiFull* and *MultiFNN*—are reconstructed on the basis of a multivariate time series which is composed of three univariate time series. Each of the series is related to movement at a given joint in one of the motion planes: sagittal, frontal, and transverse. The *MultiFull* space is built on the basis of three pairs of independently determined parameters (*m*_*k*_, *τ*_*k*_), *k*=1,2,3, resulting in space dimensionality *m*=*m*_1_+*m*_2_+*m*_3_. The average mutual information (AMI) method [30] was used in each case to determine time delays, whereas the false nearest neighbors (FNN) method was utilized to estimate embedding dimensions. The number of bins required by the AMI was determined according to the Sturges formula [31], and according to Kennel et al.'s example [22], the following values were assigned to the first (Rtol) and the second (Atol) criterion of the FNN for designating a point as a “false” neighbor: Rtol=15, Atol=2. However, in the case of *MultiFNN*, a variant of the FNN adjusted to multivariate time series was applied which takes into consideration the quota of work done during gait in individual motion planes. Based on this criterion, the planes are ordered descending as follows: sagittal, frontal, and transverse [28].

The dimensionality of *MultiFNN* space is determined holistically, i.e., on all three time series treated as a whole. Starting from 1, the embedding dimension is gradually increased by inserting successive elements to the vector of delay coordinates. The coordinates are taken from cyclically changed component time series *S*, *F*, and *T* (the symbols “*S*,” “*F*,” and “*T*” stand for sagittal, frontal, and transverse planes, respectively), with appropriate time delay (*τ*_*S*_, *τ*_*F*_, *τ*_*T*_) for each time series. The time series describing a movement in the sagittal plane is the first one used in each cycle. The stop criterion is met when the percentage of the “false” nearest neighbors falls below a given threshold (e.g. 1%) (a neighbor of a given point *P* in a space of dimensionality *d* turns out to be “false,” when it is no longer a neighbor of *P* in a space of dimensionality *d*+1). As a result, the number of coordinates taken from the *S* time series (*m*_*S*_) cannot be lower than the number of coordinates from the *F* series (*m*_*F*_) which, in turn, cannot be lower than the number of coordinates from the *T* series (*m*_*T*_): *m*_*S*_ ≥ *m*_*F*_ ≥ *m*_*T*_. By that means, the method to some extent takes into account the domination of an anterior-posterior movement in gait.

This research is a part of an extensive project carried out in cooperation with the University of the Third Age (U3A). The project focuses on elderly people that would like to remain active over the age of 65.  Table 1 presents characteristics of 14 U3A students, who agreed to participate in the experiments (12 women, 2 men), including median, mean, and standard deviation (SD) values of age, height, weight, and the body mass index (BMI).

The authors state that the study has been approved by the Ethical Committee and all the subjects gave informed written consent to participate in the research after they were briefly introduced to the research protocol.

The CAREN extended system, which was used as the research environment, guaranteed not only fully immersive virtual scenery and 6 DOF motion platform but also safety and comfort of the participants. Besides, during the experiments, the walkers were under constant medical supervision. It is worth mentioning that the U3A students willingly took part in the experiments, especially when the research environment turned out to be so immersive, attractive, and safe at the same time, as the CAREN extended system is.

The participants performed six scenarios of self-paced or fixed speed treadmill walking on level ground or on inclined platform, which are briefly presented in Table 2.

In each scenario, the subjects walked through a virtual forest. The CAREN treadmill's self-paced mode enables the subject to initiate gait and walk at her/his own pace which determines the instant walking speed. The treadmill adjusts then its speed to adapt to the subjects' pace. The self-paced mode was used in *Normal*, *Perturbation*, *Up*, and *Down* scenarios. The CAREN output data include the instant walking speed, so the values referring to the *Normal* scenario were averaged, thereby determining the basis ($\bar{{\text{PWS}}_{\text{s}}}$—subject's mean preferred walking speed) for the imposed constant walking speed used in *Faster* and *Slower* scenarios ($1.2\cdot \bar{{\text{PWS}}_{\text{s}}}$ and $0.8\cdot \bar{{\text{PWS}}_{\text{s}}}$, respectively). A single external disruption used in the *Perturbation* scenario was a sudden vertical jerk of a platform with a constant amplitude for all the participants. The perturbation was induced unexpectedly by the staff member, who supervised the experiment, at time instants which were similar for all the subjects.

The participants practiced each scenario until they were able to walk comfortably. Next, three trials were recorded using the integrated Vicon motion capture system at the frequency of 100 Hz giving together 18 gait sequences for every subject (several exceptions were caused by fatigue). Every time series, which is analyzed by means of the LLE, should include the equal number of strides as well as the equal number of data points [18]. So, the sequences were long enough to contain 50 strides as the assumed final length.

The recorded data were initially filtered and optionally repaired (e.g., in view of occluded markers) using the Vicon software. The beginning of each stride was demarcated based on precisely marked occurrence of the “heel-strike” event. A stride interval varied not only across subjects but also across experiments' scenarios. Mean and standard deviation values of the stride interval for different scenarios are included in Table 3.

Where necessary, the time series were cropped to 50 strides. Next, every stride was separately normalized using linear interpolation to contain 100 points. Subsequently, the time series were subject to estimation of reconstruction parameters. It deserves a mention that the most frequently occurring values for the dimensionality of the *MultiFNN* state space are 7, 8 (*UniS*: 4, 5; *MultiFull*: 14, 15). Afterward, the short-term LLE values were calculated using the reconstructed trajectory and taking into account two variants of a time span

- of a length equal to 50 which is equivalent to one step, i.e., a half of a stride (the short-term LLE is then labeled by *λ*_*S*0.5_)
- of a length equal to 100 which is equivalent to one stride (*λ*_*S*1_)

The mean period parameter as the threshold for temporal separation of the nearest neighbors on two different segments of the reconstructed state space trajectory, which repeatedly imitate two initially neighboring trajectories, was estimated as the reciprocal of the mean frequency of the power spectrum [7]. In each iteration, one segment starts from the next point of the reconstructed trajectory. Taking into account the temporal separation mentioned above, the nearest neighbor of this starting point on the adjacent orbit becomes the first point of the second segment. The length of the segments was set to 1000. The Euclidean distance for each pair of two corresponding points on both segments is computed and stored to finally determine the average logarithmic divergence of the neighboring trajectories which is required to estimate the LLE value.

Finally, the results were aggregated across all the six scenarios, three state space structures, and individual joints. All the computations were performed using MATLAB and MySQL DBMS. The statistical analysis of the results focused on investigating if differences between the short-term LLE values in three pairs of compared scenarios (*Normal*-*Perturbation*, *Faster*-*Slower*, *Up*-*Down*) are significant for individual joints, taking into account that the short-term LLE values were computed using different state space structures. The complete analysis was performed in Excel using the Real Statistics Resource Pack (https://www.real-statistics.com/).

## 3. Results

**Figure 1:**
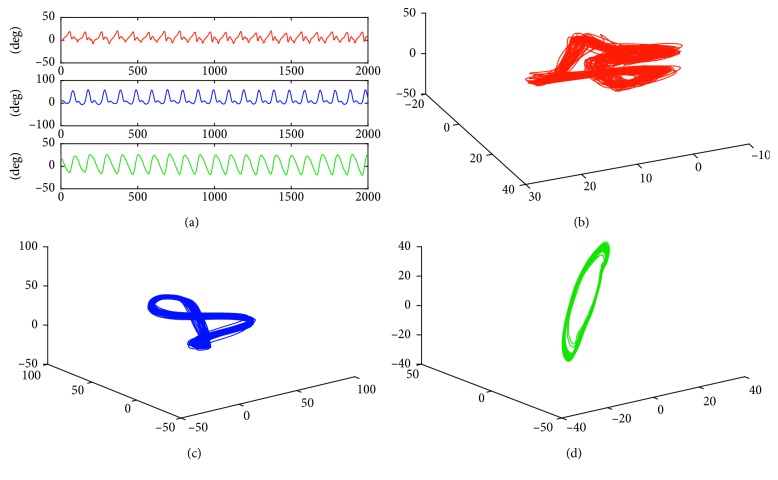
(a) Time series representing a movement in sagittal plane (red line: left ankle joint, blue line: left knee joint, and green line: left hip joint); (b–d) 3D projections of corresponding reconstructed trajectories for: (b) left ankle joint; (c) left knee joint; (d) left hip joint. Each of the examined joints is characterized by a specific shape of the trajectory.

Examples of times series representing a movement in the sagittal plane, recorded for a 75-year-old woman performing the *Normal* scenario are presented in Figure 1(a) (the red line corresponds to the left ankle joint, the blue line to the left knee joint, and the green one to the left hip joint). Each of these series was independently used for trajectory reconstruction in *UniS* state space. The corresponding 3D projections of the reconstructed trajectories are presented in Figure 1(b) (left ankle joint), Figure 1(c) (left knee joint), and Figure 1(d) (left hip joint).

**Figure 2:**
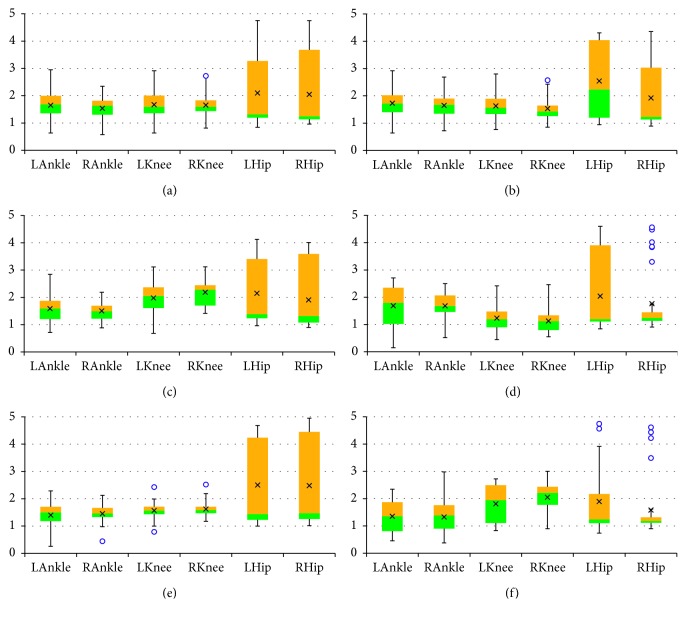
Box plots for *λ*_*S*0.5_ computed in *UniS* state space for different scenarios: (a) *Normal*; (b) *Perturbation*; (c) *Faster*; (d) *Slower*; (e) *Up*; (f) *Down*. All values are positive. The platform jerks seem to have no effect. The walking speed seems to affect the knee joint primarily, while the platform inclination seems to have the greatest impact on the hip joint.

**Figure 3:**
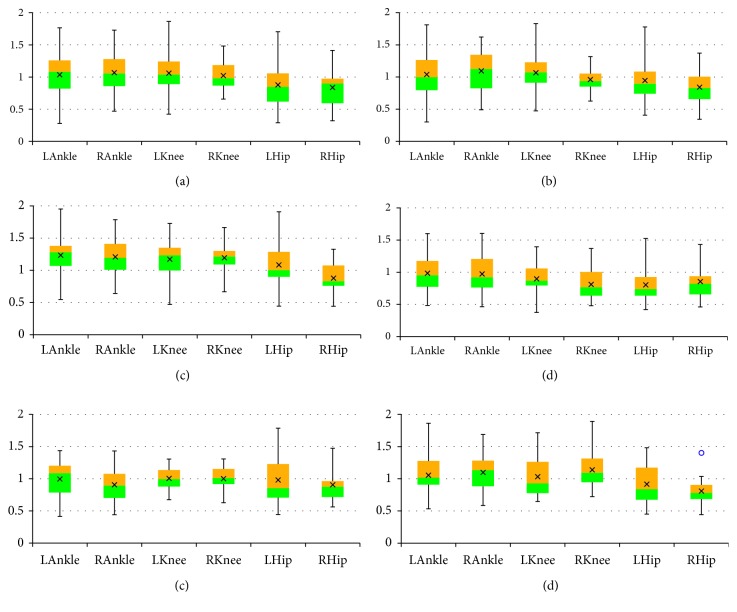
Box plots for *λ*_*S*0.5_ computed in *MultiFull* state space for different scenarios: (a) *Normal*; (b) *Perturbation*; (c) *Faster*; (d) *Slower*; (e) *Up*; (f) *Down*. All values are positive. The platform jerks seem to have no effect. The walking speed still seems to affect the knee joint in the first place, while the impact of the platform inclination on the hip joint is no longer noticeable.

**Figure 4:**
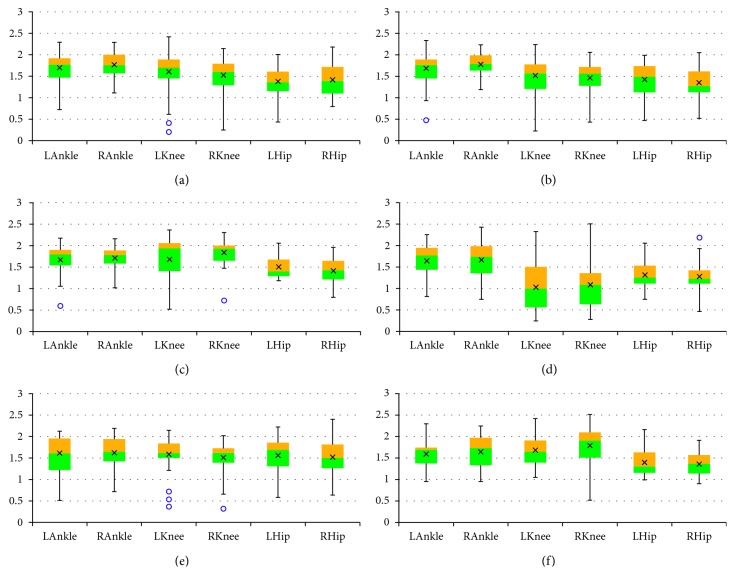
Box plots for *λ*_*S*0.5_ computed in *MultiFNN* state space for different scenarios: (a) *Normal*; (b) *Perturbation*; (c) *Faster*; (d) *Slower*; (e) *Up*; (f) *Down*. All values are positive. The platform jerks seem to have no effect. The walking speed seems to affect the knee joint primarily, while the platform inclination seems to have the greatest impact on the hip joint.

**Figure 5:**
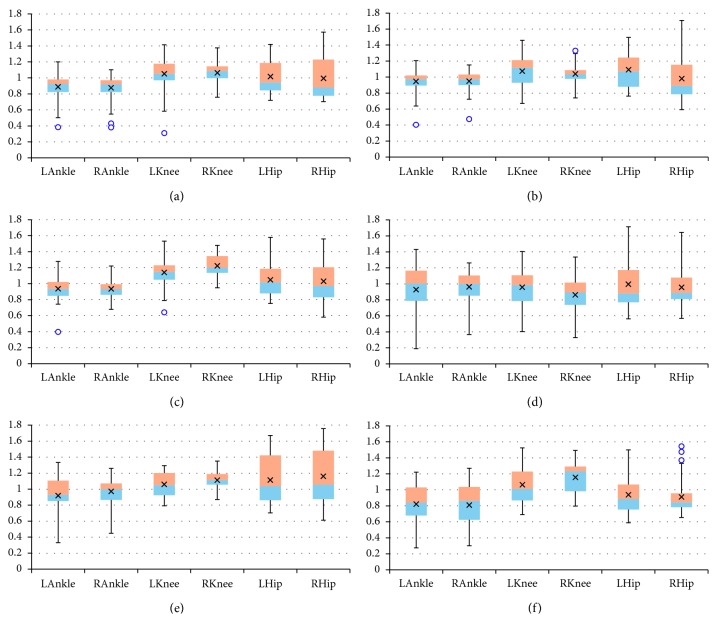
Box plots for *λ*_*S*1_ computed in *UniS* state space for different scenarios: (a) *Normal*; (b) *Perturbation*; (c) *Faster*; (d) *Slower*; (e) *Up*; (f) *Down*. All values are positive but smaller than the corresponding *λ*_*S*0.5_ values: locally unstable behavior is gradually attenuated by the locomotor system. The platform jerks seem to have no effect. The walking speed seems to affect the knee joint primarily, while the platform inclination seems to have the greatest impact on the hip joint.

**Figure 6:**
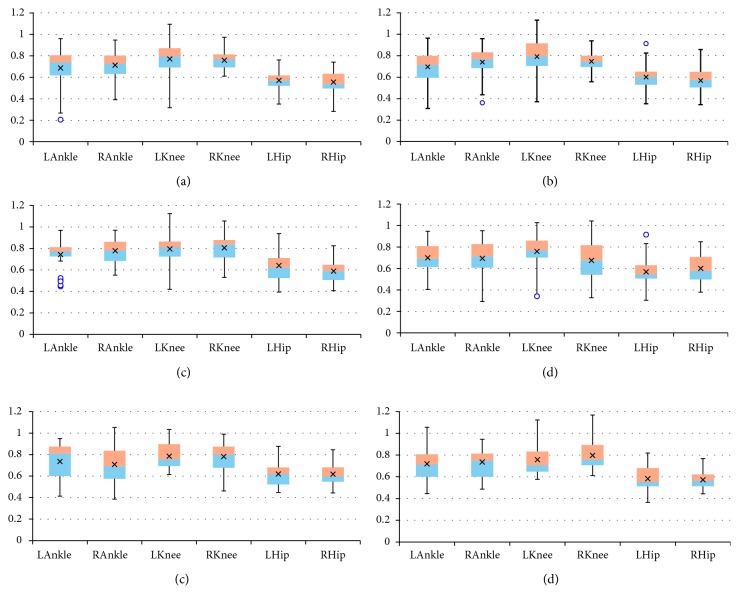
Box plots for *λ*_*S*1_ computed in *MultiFull* state space for different scenarios: (a) *Normal*; (b) *Perturbation*; (c) *Faster*; (d) *Slower*; (e) *Up*; (f) *Down*. All values are positive but smaller than the corresponding *λ*_*S*0.5_ values: locally unstable behavior is gradually attenuated by the locomotor system. The platform jerks seem to have no effect. The previously indicated dependencies for individual joints are no longer visible.

**Figure 7:**
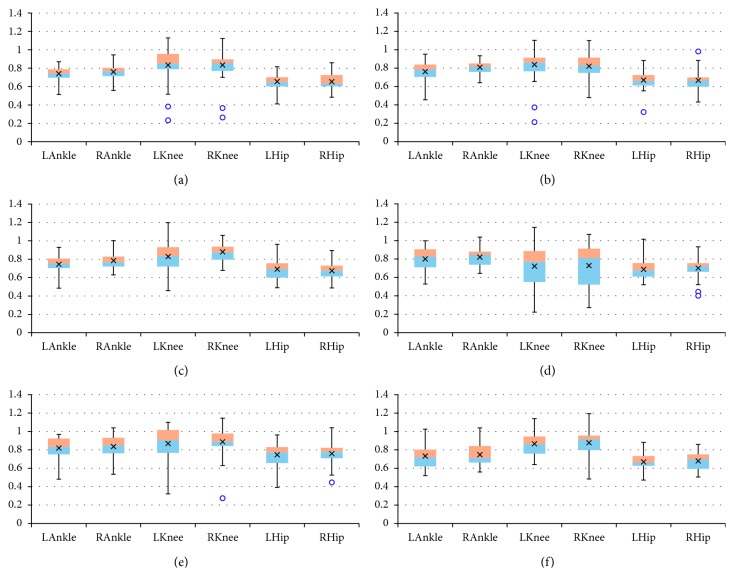
Box plots for *λ*_*S*1_ computed in *MultiFNN* state space for different scenarios: (a) *Normal*; (b) *Perturbation*; (c) *Faster*; (d) *Slower*; (e) *Up*; (f) *Down*. All values are positive but smaller than the corresponding *λ*_*S*0.5_ values: locally unstable behavior is gradually attenuated by the locomotor system. The platform jerks seem to have no effect. The walking speed seems to affect the knee joint primarily, while the platform inclination seems to have the greatest impact on the hip joint.

The final results are presented as box plots. On each box, the boundary between the areas of different colors indicates the median, the × symbol denotes the mean, the edges of the box are the 25^th^ and 75^th^ percentiles, the “whiskers” indicate the most extreme values which are not outliers, i.e., the smallest value that is larger than or equal to *Q*_1_ – 1.5 · (*Q*_3_ − *Q*_1_) and the largest value that is less than or equal to *Q*_3_+1.5 · (*Q*_3_ − *Q*_1_), where *Q*_1_ and *Q*_3_ denote the 25^th^ and 75^th^ percentiles, respectively, and the outliers are individually marked by circles. Figures 2–4 include box plots for *λ*_*S*0.5_ computed in *UniS*, *MultiFull*, and *MultiFNN* spaces, respectively. Analogically aggregated results for *λ*_*S*1_ are presented in Figures 5–7. Each figure comprises six subfigures corresponding to six trial scenarios: (a) *Normal*, (b) *Perturbation*, (c) *Faster*, (d) *Slower*, (e) *Up*, and (f) *Down*. Each subfigure includes separate box plots for ankle, knee, and hip joints on both sides of a body (“L,” left; “R,” right).

## 4. Discussion

Some general remarks, which were formulated at the earlier stage of research [29], will be briefly reminded here for clarity of further considerations. First, it is worth mentioning that the positive values of both short-term LLE variants calculated for a uni- or multivariate time series utilizing each of the considered state spaces confirm that the elderly are locally unstable during gait; however, the LLE values are lower than our results for young subjects reported in [24]. Besides, the relationship between the corresponding LLE values (*λ*_*S*0.5_ > *λ*_*S*1_ > 0) indicates that locally unstable behavior is gradually attenuated by the locomotor system. Secondly, comparisons between the *Faster* and *Slower* scenarios (e.g., in Figures 2(c) and 2(d), 3(c) and 3(d), 4(c) and 4(d), 5(c) and 5(d)) as well as between *Up* and *Down* scenarios (e.g., in Figures 2(e) and 2(f), 4(e) and 4(f)) suggest that both walking slower and walking downwards are more stable than their opposed variants.

The main goal of the statistical analysis is to investigate if the differences between the opposed scenarios for individual joints are significant, taking into consideration that the short-term LLE values were computed in two variants using different state space structures.

For a given joint *J* (ankle, knee, and hip), measure *M* (*λ*_*S*0.5_ and *λ*_*S*1_), state space *S* (*UniS*, *MultiFull*, and *MultiFNN*), and the pair of opposed scenarios *Sc*_1_ and *Sc*_2_ (*Normal*-*Perturbation*, *Faster-Slower*, and *Up-Down*), a null hypothesis *H*_0_(*J*, *M*, *S*) is formulated as follows: *P*(*Sc*_1_ > *Sc*_2_)=*P*(*Sc*_2_ > *Sc*_1_). The null hypothesis assumes that with respect to joint *J*, measure *M*, and state space *S*, the probability of an observation randomly selected from the group related to the *Sc*_1_ scenario exceeding an observation randomly selected from the group representing the *Sc*_2_ scenario equals the probability of an observation randomly selected from the group related to the *Sc*_2_ exceeding an observation randomly selected from the group representing the *Sc*_1_.

The expected conclusions are placed in the alternative hypotheses, according to which observations in one group tend to be greater than observations in the other group, e.g., *H*_1_(knee, *λ*_*S*0.5_, *UniS*): *P*(*Faster* > *Slower*) > *P*(*Slower* > *Faster*) (see Figures 2(c) and 2(d)).

The selection of an appropriate statistical test for verification of hypotheses should be preceded by the analysis of distribution for both measures *λ*_*S*0.5_ and *λ*_*S*1_ independently, taking into account each dataset related to a pair (scenario, joint) separately. Totally, 216 cases were considered (3 joints *∗* 2 body sides *∗* 6 scenarios *∗* 3 state spaces *∗* 2 measures = 216 datasets).

The outcome of the Shapiro–Wilk test of normality at significance level of 5% turned out to be negative in 74 from 216 cases (ca. 34%). Thus, the verification of the hypotheses requires a nonparametric test without any assumptions related to the distribution of scores.

With regard to the outcomes of the Shapiro–Wilk test, the hypotheses were verified for both measures independently using the nonparametric Mann–Whitney–Wilcoxon test at significance level of 5%. Because the number of null hypotheses is large (3 joints *∗* 3 pairs of scenarios *∗* 3 state spaces *∗* 2 measures = 54), the results of their verification are presented symbolically. Each of Tables 4–9 represents a pair (measure *M*, state space *S*) and includes “=” if for a given pair of scenarios *Sc*_1_, *Sc*_2_ and a given joint *J* the null hypothesis *H*_0_(*J*, *M*, *S*): *P*(*Sc*_1_ > *Sc*_2_)=*P*(*Sc*_2_ > *Sc*_1_) was not rejected. Otherwise, one of the symbols “<,” “>” determines a one-sided alternative hypothesis (*H*_1_(*J*, *M*, *S*): *P*(*Sc*_1_ > *Sc*_2_) < *P*(*Sc*_2_ > *Sc*_1_) or *H*_1_(*J*, *M*, *S*): *P*(*Sc*_1_ > *Sc*_2_) > *P*(*Sc*_2_ > *Sc*_1_)), in favor of which the corresponding null hypothesis was rejected.

The total number of rejected null hypotheses for individual pairs of scenarios is as follows: *Normal*-*Perturbation*: 2, *Faster*-*Slower*: 11, and *Up*-*Down*: 10. Thus, there are no significant differences in the local dynamic stability between *Normal* and *Perturbation* scenarios. The platform jerks were probably too mild and too infrequent to have an impact on the short-term LLE.

As far as two other pairs of scenarios are concerned, the high number of rejected null hypotheses indicates that the sensitivity to tiny local perturbations in both compared scenarios is different. The lower values of the short-term LLE for the *Slower* scenario suggest that this variant of walking is conducive to greater stability and this observation is consistent with a common impression that elderly people, scared of fall, try to walk more carefully. During slow gait, the movement of limbs is more accurately controlled by the central nervous system.

Independently of the LDS measure and the applied state space, the significant differences between *Faster* and *Slower* scenarios are always visible in the case of a knee joint. LDS for the knee joint is lower in faster walking due to more frequent loss of body balance, more frequent bending of the knee, and the largest lateral knee movements.

A similar remark refers to a hip joint as regards the *Up*-*Down* pair where the hip joint is responsible for bending and straightening the torso. The lower values of the short-term LLE for the *Down* scenario are supposedly caused by a slightly rigid way of walking downwards and involuntary straightening.

The lower values for hip joints for *MultiFull* and *MultiFNN* spaces in comparison to *UniS* space result from using a multivariate time series and thus adding information about movement in frontal and transverse planes.

The verification outcomes for *UniS* and *MultiFNN* spaces are very consistent (in 8 from 9 cases for each measure). It can be explained by the domination of movement in the sagittal plane during gait. The *MultiFNN* space is based only on a subset of delay coordinates from a multivariate time series as opposed to the *MultiFull* space which includes all of them. Consequently, in the case of *MultiFNN* space, the delay coordinates, which were determined on the basis of a component time series describing a movement at a joint in the sagittal plane, play a predominant role. In both cases, in which the result of verification is different for *UniS* and *MultiFNN* ({hip, *λ*_*S*0.5_, *Faster-Slower*}, {ankle, *λ*_*S*1_, *Faster-Slower*}), the null hypothesis was not rejected for *UniS*, while it happened for *MultiFNN* which was presumably caused by additional information related to other motion planes. Greater discrepancies between *UniS* and *MultiFull* (7 different verification results) as well as between *MultiFNN* and *MultiFull* (5 different verification results) suggest that *MultiFull* includes redundant information.

Inspired by the close connection between *UniS* and *MultiFNN*, the authors conducted next tests to assess a statistical significance of differences between datasets related to individual state spaces (for *λ*_*S*0.5_ and *λ*_*S*1_ separately). The deviation of individual datasets from normal distributions and the lack of homogeneity of their variances were showed by the Shapiro–Wilk test and the Levene test, respectively. In consequence, a significant difference between all three datasets was identified by means of the nonparametric Kruskal–Wallis test. Afterward, the Nemenyi test and the pairwise Mann–Whitney test indicated that the following pairs of datasets: *UniS*-*MultiFull* and *MultiFull*-*MultiFNN* are significantly different and confirmed that the pair *UniS*-*MultiFNN* is not significantly different; however, only in the case of *λ*_*S*0.5_ (*p* values were 0.99 for the Nemenyi test and 0.50 for the pairwise Mann–Whitney test). The risk of committing a type I error was reduced by using the Bonferroni correction.

Therefore, although each examined state space reveals locally unstable behavior during gait as well as its attenuation in the current stride, a direct comparison of the short-term Lyapunov exponents computed for different state spaces bears the risk of a wrong conclusion.

A similar remark refers to another test in which the accepted hypotheses based on *λ*_*S*0.5_ were juxtaposed with their counterparts based on *λ*_*S*1_. In 16 of 27 (ca. 59%) cases (where each case was determined by a joint and a state space), the hypotheses were consistent. It means that the length of a time span, over which the short-term LLE is computed, must be taken into account when comparing the results of different studies.

It should also be noted that the structure of a state space affects the computation time which comprises the following operations on a uni- or multivariate time series: interpolation, estimation of time delay(s) and embedding dimension(s), reconstruction of the state space trajectory, and computation of the short-term LLE. The mean computation time values for individual state spaces are presented in Table 10. The computer parameters were as follows: Aspire X5950, Intel Core i5, 3.2 GHz, RAM 4 GB.

As expected, the mean computation time is maximal for the *MultiFull* space (owing to the estimation of three embedding dimensions) and almost three times smaller for the *MultiFNN* space (embedding dimension was estimated only once). The mean computation time for the *MultiFNN* space turned out to be even slightly smaller than in the case of the *UniS* which is based on a univariate time series.

It is worth mentioning that some other studies which require the LLE computation and are based on a univariate time series could benefit from adding one or more time series to input data used for a state space construction. For instance, dynamic features of eye movement [32] were originally analyzed only using the values of the first derivative of horizontal eye displacements during a fixation. A two-dimensional time series could be created which would also include velocity in the vertical direction while maintaining the dominant role of horizontal eye displacements. Another example is motion data-based quality assessment of completion of rehabilitation exercises maintaining mobility of the hip in case of coxarthrosis [33] where the complex procedure of the LLE computation using a multivariate time series could replace or expand the results of the RQA (recurrence quantification analysis) measures' application for a time series describing a hip joint movement in the sagittal plane.

## 5. Conclusions

The method of computation of the local dynamic stability requires a proper reconstruction of a state space trajectory. The authors intended to investigate how a state space structure affects the short-term LLE as the measure related to the local dynamic stability. One of the state spaces was constructed using an algorithm which estimates the embedding dimension holistically in the case of a multivariate gait time series while taking into account the quota of work done during gait in individual motion planes. It should also be mentioned here that a direct comparison of the short-term LLE computed for different state spaces is burdened with the risk of a wrong conclusion. Due attention should also be paid to the length of the time span, over which the short-term LLE was estimated.

The improved method of LDS assessment will be used in experiments focused on finding easy-to-measure, objective biomarkers that could classify PD (Parkinson's disease) patients in early (preclinical) stages of the disease. Identification of the first deviation from the norm in patient's physical movement like walking, which is often unobservable to a neurologist, might help follow disease progression, make more adjusted treatment, and lead to modification of disease course.

## Figures and Tables

**Table 1:** Participants' characteristics.

	Age (years)	Height (m)	Weight (kg)	BMI (kg/m^2^)
Median	71	1.65	70	25.86
Mean ± SD	70.64 ± 3.52	1.66 ± 0.07	76.12 ± 15.57	27.66 ± 5.18

**Table 2:** Scenarios of experiments.

Scenario	Type of walking	Platform slope (°)
*Normal*	Self-paced	0
*Perturbation*	Self-paced	0
*Faster*	At fixed treadmill speed $\left(1.2\cdot \bar{{\text{PWS}}_{\text{s}}}\right)$	0
*Slower*	At fixed treadmill speed $\left(0.8\cdot \bar{{\text{PWS}}_{\text{s}}}\right)$	0
*Up*	Self-paced	+5
*Down*	Self-paced	−5

**Table 3:** Mean stride interval values for six scenarios (s).

	*Normal*	*Perturbation*	*Faster*	*Slower*	*Up*	*Down*
Mean	1.0924	1.0853	0.9590	1.4226	1.2316	1.0079
SD	0.1085	0.1063	0.0998	0.2746	0.1537	0.1222

**Table 4:** Results of verification of statistical hypotheses for *λ*_*S*0.5_ and *UniS*.

	*Normal-Perturbation*	*Faster-Slower*	*Up-Down*
Ankle joint	=	=	=
Knee joint	=	>	<
Hip joint	=	=	>

**Table 5:** Results of verification of statistical hypotheses for *λ*_*S*1_ and *UniS*.

	*Normal-Perturbation*	*Faster-Slower*	*Up-Down*
Ankle joint	<	=	>
Knee joint	=	>	=
Hip joint	=	=	>

**Table 6:** Results of verification of statistical hypotheses for *λ*_*S*0.5_ and *MultiFull*.

	*Normal-Perturbation*	*Faster-Slower*	*Up-Down*
Ankle joint	=	>	>
Knee joint	=	>	=
Hip joint	=	>	=

**Table 7:** Results of verification of statistical hypotheses for *λ*_*S*1_ and *MultiFull*.

	*Normal-Perturbation*	*Faster-Slower*	*Up-Down*
Ankle joint	=	>	=
Knee joint	=	>	=
Hip joint	=	=	>

**Table 8:** Results of verification of statistical hypotheses for *λ*_*S*0.5_ and *MultiFNN*.

	*Normal-Perturbation*	*Faster-Slower*	*Up-Down*
Ankle joint	=	=	=
Knee joint	=	>	<
Hip joint	=	>	>

**Table 9:** Results of verification of statistical hypotheses for *λ*_*S*1_ and *MultiFNN*.

	*Normal-Perturbation*	*Faster-Slower*	*Up-Down*
Ankle joint	<	<	>
Knee joint	=	>	=
Hip joint	=	=	>

**Table 10:** Mean computation time values for individual state spaces (s).

*UniS*	*MultiFull*	*MultiFNN*
30.96	80.89	28.30

## Data Availability

The motion capture data (joint angles) used to support the findings of this study are available from the corresponding author upon request.

## References

[1] Dingwell J. B., Cusumano J. P. (2000). Nonlinear time series analysis of normal and pathological human walking. *Chaos: An Interdisciplinary Journal of Nonlinear Science*.

[2] Hamacher D., Hamacher D., Singh N. B., Taylor W. R., Schega L. (2015). Towards the assessment of local dynamic stability of level-grounded walking in an older population. *Medical Engineering & Physics*.

[3] Hamacher D., Hamacher D., Rehfeld K., Schega L. (2016). Motor-cognitive dual-task training improves local dynamic stability of normal walking in older individuals. *Clinical Biomechanics*.

[4] van Schooten K. S., Rispens S. M., Pijnappels M., Daffertshofer A., van Dieën J. H. (2013). Assessing gait stability: the influence of state space reconstruction on inter- and intra-day reliability of local dynamic stability during over-ground walking. *Journal of Biomechanics*.

[5] Dingwell J. B., Marin L. C. (2006). Kinematic variability and local dynamic stability of upper body motions when walking at different speeds. *Journal of Biomechanics*.

[6] England S. A., Granata K. P. (2007). The influence of gait speed on local dynamic stability of walking. *Gait & Posture*.

[7] Rosenstein M. T., Collins J. J., De Luca C. J. (1993). A practical method for calculating largest Lyapunov exponents from small data sets. *Physica D: Nonlinear Phenomena*.

[8] Perc M. (2005). The dynamics of human gait. *European Journal of Physics*.

[9] Rosenstein M. T., Collins J. J., De Luca C. J. (1994). Reconstruction expansion as a geometry-based framework for choosing proper delay times. *Physica D: Nonlinear Phenomena*.

[10] Vlachos I., Kugiumtzis D. (2008). State space reconstruction for multivariate time series prediction. *Nonlinear Phenomena in Complex Systems*.

[11] Cao L., Mees A., Judd K. (1998). Dynamics from multivariate time series. *Physica D: Nonlinear Phenomena*.

[12] Terrier P., Luthi F., Deriaz O. (2013). Do orthopaedic shoes improve local stability of gait? An observation study in patients with chronic foot and ankle injuries. *BMC Musculoskeletal Disorders*.

[13] Bruijn S. M., Meijer O. G., Beek P. J., van Dieën J. H. (2010). The effects of arm swing on human gait stability. *Journal of Experimental Biology*.

[14] Toebes M. J. P., Hoozemans M. J. M., Furrer R., Dekker J., van Dieën J. H. (2012). Local dynamic stability and variability of gait are associated with fall history in elderly subjects. *Gait & Posture*.

[15] Reynard F., Vuadens P., Deriaz O., Terrier P. (2014). Could local dynamic stability serve as an early predictor of falls in patients with moderate neurological gait disorders? a reliability and comparison study in healthy individuals and in patients with paresis of the lower extremities. *PLoS One*.

[16] Lockhart T. E., Liu J. (2008). Differentiating fall-prone and healthy adults using local dynamic stability. *Ergonomics*.

[17] Hamacher D., Singh N. B., van Dieën J. H., Heller M. O., Taylor W. R. (2011). Kinematic measures for assessing gait stability in elderly individuals: a systematic review. *Journal of the Royal Society Interface*.

[18] Bruijn S. M., Meijer O. G., Beek P. J., van Dieёn J. H. (2013). Assessing the stability of human locomotion: a review of current measures. *Journal of the Royal Society Interface*.

[19] van Emmerik R. E. A., Ducharme S. W., Amado A. C., Hamill J. (2016). Comparing dynamical systems concepts and techniques for biomechanical analysis. *Journal of Sport and Health Science*.

[20] Gates D. H., Dingwell J. B. (2009). Comparison of different state space definitions for local dynamic stability analyses. *Journal of Biomechanics*.

[21] Liu K., Wang H., Xiao J. (2015). The multivariate largest Lyapunov exponent as an age-related metric of quiet standing balance. *Computational and Mathematical Methods in Medicine*.

[22] Kennel M. B., Brown R., Abarbanel H. D. I. (1992). Determining embedding dimension for phase-space reconstruction using a geometrical construction. *Physical Review A*.

[23] Zhang C.-T., Guo J., Ma Q.-L., Peng H., Zhang X.-D. Phase space reconstruction and prediction of multivariate chaotic time series.

[24] Piórek M., Josiński H., Michalczuk A., Świtoński A., Szczęsna A. (2017). Quaternions and joint angles in an analysis of local stability of gait for different variants of walking speed and treadmill slope. *Information Sciences*.

[25] Szczęsna A. (2019). Quaternion entropy for analysis of gait data. *Entropy*.

[26] Mehdizadeh S. (2018). The largest Lyapunov exponent of gait in young and elderly individuals: a systematic review. *Gait & Posture*.

[27] Jagrič T., Marhl M., Štajer D. (2007). Irregularity test for very short electrocardiogram (ECG) signals as a method for predicting a successful defibrillation in patients with ventricular fibrillation. *Translational Research*.

[28] Eng J. J., Winter D. A. (1995). Kinetic analysis of the lower limbs during walking: what information can be gained from a three-dimensional model?. *Journal of Biomechanics*.

[29] Josiński H., Świtoński A., Michalczuk A., Grabiec P., Pawlyta M., Wojciechowski K. Analysis of chaotic behaviors in gait of the elderly using the CAREN extended system.

[30] Henry B., Lovell N., Camacho F. (2001). Nonlinear dynamics time series analysis. *Nonlinear Biomedical Signal Processing: Dynamic Analysis and Modeling*.

[31] Sturges H. A. (1926). The choice of a class interval. *Journal of the American Statistical Association*.

[32] Harężlak K. (2017). Eye movement dynamics during imposed fixations. *Information Sciences*.

[33] Josiński H., Michalczuk A., Mucha R., Świtoński A., Szczęsna A., Wojciechowski K. Analysis of human motion data using recurrence plots and recurrence quantification measures.

